# Aligning statistical models with inference goals in the neuroscience of language: A dual-dependency taxonomy

**DOI:** 10.1162/IMAG.a.1132

**Published:** 2026-02-17

**Authors:** Sophie Bouton, Valérian Chambon, Narly Golestani, Elia Formisano, Timothée Proix, Anne-Lise Giraud

**Affiliations:** Université Paris Cité, Institut Pasteur, AP-HP, Inserm, Fondation Pour l’Audition, Institut de l’Audition, IHU reConnect, Paris, France; Laboratoire de Sciences Cognitives et Psycholinguistique, Département d’Etudes Cognitives, Ecole Normale Supérieure, EHESS, CNRS, PSL University, Paris, France; Institut Jean Nicod, CNRS/École Normale Supérieure UMR 8129, PSL University, Paris, France; Section of Psychology, University of Geneva – Campus Biotech, Geneva, Switzerland; Brain and Language Lab, Vienna Cognitive Science Hub, University of Vienna, Vienna, Austria; Department of Behavioral and Cognitive Biology, Faculty of Life Sciences, University of Vienna, Vienna, Austria; Department of Cognitive Neuroscience, Faculty of Psychology and Neuroscience, Maastricht University, Maastricht, the Netherlands; Maastricht Brain Imaging Center, Maastricht, the Netherlands; Maastricht Centre for Systems Biology, Maastricht University, Maastricht, the Netherlands; Department of Basic Neurosciences, University of Geneva – Campus Biotech, Geneva, Switzerland

**Keywords:** data analysis, interpretation, dependency, features, language

## Abstract

Language unfolds over time and across multiple representational levels, from acoustics to meaning. Neural systems must, therefore, integrate temporal with representational structure, linking the evolving input with the hierarchical units it instantiates. These operations give rise to two fundamental statistical dependencies in linguistic and neural data: covariance, the instantaneous shared structure across features or recording sites, and temporal dependence, the influence of past states on the present state. As experiments become more naturalistic, neuroimaging and electrophysiological data increasingly express both forms of structure, producing correlated variables and continuous temporal dependencies that complicate interpretation. Because statistical models handle covariance and temporal dependence differently, they support distinct kinds of inference about language-brain mapping. We introduce a Dual-Dependency Taxonomy that classifies modeling approaches by the dependencies they represent. This framework clarifies the linguistic-neural relationships each model family can reveal, the questions they cannot address, and the methodological implications that follow.

## 1. Interpretability in Neural Modeling

Language draws on general neural circuits that anticipate, integrate, and update information (Buckner & Krienen, 2013; Kikuchi et al., 2018; Marslen-Wilson & Bozic, 2018). It is processed on multiple timescales, from the rapid succession of phonemes to the slower integration of words and meaning and engages widely distributed brain regions that cooperate to extract, predict, and combine linguistic information. This coordination allows the brain to continuously map variable acoustic signals onto meaningful units despite uncertainty and noise in the input. As experiments become more naturalistic, neuroimaging and electrophysiological recordings have grown richer, and the core difficulty is no longer a shortage of data but the rich correlation within them: multiple linguistic and neural variables co-vary and evolve in parallel, challenging simple cause-and-effect interpretations. In such settings, modeling choices become central, because different statistical approaches make different aspects of this structured variability explicit. Each type of model, therefore, supports a distinct kind of inference about how language is represented and computed in the brain. Choosing among them is not merely a technical matter: it determines what we can legitimately conclude about the underlying computations. To make these differences explicit, we introduce a Dual-Dependency Taxonomy (2D-Taxonomy) that organizes neural models by how they handle covariance (shared structure at a moment in time) and temporal dependence (the influence of the past on the present). By linking model assumptions to the dependencies they encode, this framework clarifies the trade-offs between predictive fit and interpretability and delineates the inferential space each model family supports.

### 1.1 From interpretability by design to statistical modeling

**Fig. 1.**
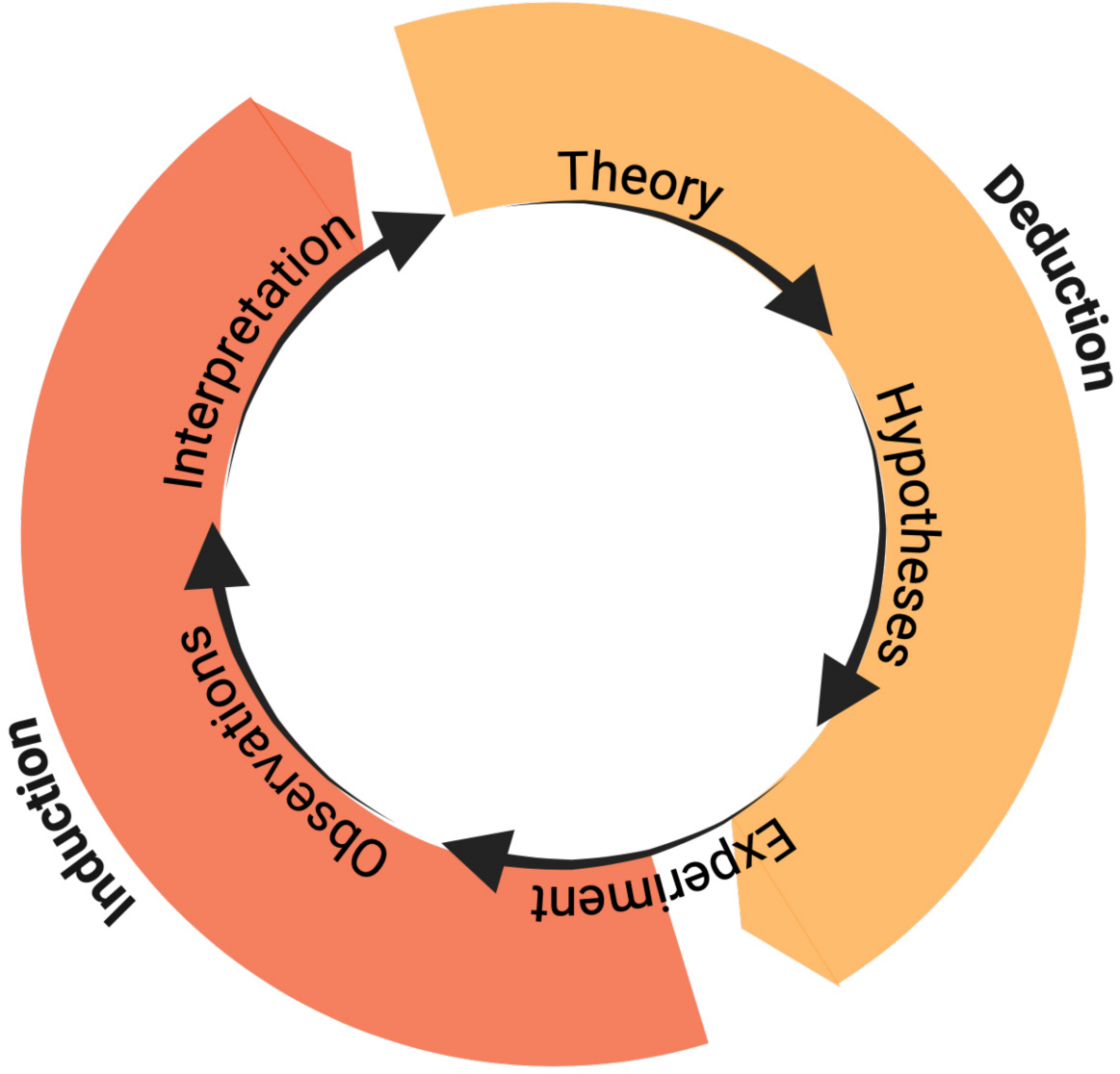
The inductive/deductive cycle illustrates the iterative logic of scientific inference: theoretical hypotheses guide experimental design (deduction), while observed patterns motivate the model refinement and the formulation of new hypotheses (induction). This cycle supports both controlled experiments and data-driven approaches.

Traditional approaches in the neuroscience of language are rooted in controlled experimentation and the logic of inductive-deductive inference (Popper, 1935; Sun, 2023). Models are refined over time through an iterative cycle of hypothesis formulation, pattern identification in neural (or neurological) data, and targeted testing (Fig. 1). This approach, including lesion studies (Bouton et al., 2018; Hickok, 2009; Mirman et al., 2015; Na et al., 2022), cortical stimulation (Long et al., 2016; Lu et al., 2021; Marchesotti et al., 2020), and tightly structured behavioral paradigms, achieves interpretability by design: the deliberate manipulation of experimental parameters allows neural activity to be directly attributed to specific linguistic processes. This logic establishes foundational links between brain regions and functions: for instance, the role of Broca’s area in speech production (Broca, 1861; Flinker et al., 2015; Pracar et al., 2025) or the anterior temporal cortex in semantic interpretation (Binney et al., 2010; Schwartz et al., 2009).

Multivariable models such as General Linear Models (GLM) extend this logic statistically. Each predictor is assumed to contribute independently to the observed neural signal, enabling clear attribution of function. This framework produces robust and replicable results: low-level acoustic manipulations engage primary auditory areas (Giroud et al., 2024; Zaehle et al., 2004), while higher-order linguistic properties such as lexical frequency or syntactic complexity elicit graded activation along the superior temporal and inferior frontal cortices (Obleser et al., 2011; Uddén et al., 2022). These findings support the view of a hierarchical organization of speech and language processing (Davis & Johnsrude, 2003; Ding et al., 2016; Overath & Paik, 2021). Yet, the interpretive clarity of these models depends on the independence of predictors—a condition that is rarely met in natural language, where acoustic, lexical, and semantic cues overlap continuously. As a result, this deductive framework, while powerful, is increasingly criticized for limited ecological validity (Hamilton & Huth, 2018).

### 1.2 The rise of complex and naturalistic modeling

To capture the richness of language as it is used and perceived, recent work has shifted toward inductive, data-driven approaches. Advances in high-density recordings and machine learning have made it possible to analyze neural responses recorded during naturalistic language experiences—such as conversation, storytelling, or spontaneous listening (Hamilton & Huth, 2018). These methods embrace correlated and overlapping features instead of controlling them away, uncovering latent structure in high-dimensional data. However, with this gain in flexibility comes a cost to interpretability. When models rely on large sets of correlated predictors, or when their internal structure is opaque, as in deep or non-linear architecture, it becomes difficult to map internal states back to cognitive operations or brain mechanisms (Whiteway & Butts, 2019).

The resulting diversity of analytical strategies has produced a fragmented landscape in which similar data can be modeled in incompatible ways, each supporting different types of claims. Some approaches emphasize unique, condition-specific effects; others seek to capture shared variance. Still others focus on temporal prediction or on reconstructing internal trajectories in latent spaces. To navigate this complexity and to make model choice itself an object of reflection, we propose a principled framework that relates modeling assumptions to the types of dependencies they represent and the inferences they allow.

## 2 A Framework Grounded in Two Statistical Dependencies

**Fig. 2.**
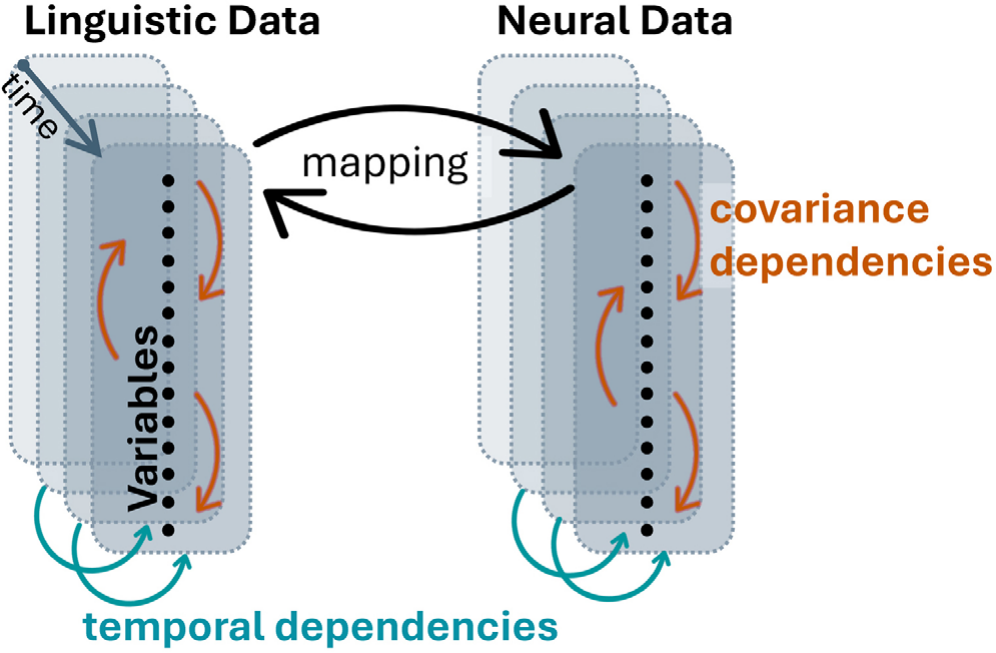
Illustration of the two core statistical dependencies in language-brain mapping: covariance dependencies (orange curved arrows) reflect correlations between co-occurring features—such as acoustic properties linked to lexical or semantic content, or spatial correlation in neural signals; temporal dependencies (blue loops), reflect how past states—phonemes, words, or neural activity—shape present responses through predictive and memory-related mechanisms. Linguistic and neural data are represented as vertically stacked variables (black dots) sampled over time (transparent layers), with mapping arrows representing statistical models linking stimulus and response.

Modeling the mapping between language and neural activity requires specifying the forms of statistical structure that models are designed to represent. In speech and language neuroscience, two such forms are fundamental (Fig. 2). The first is **covariance**: linguistic features tend to co-occur, and neural sites tend to co-activate, producing instantaneous correlations across variables. These correlations arise from different sources, such as the natural co-occurrence of acoustic intensity, spectral content, and lexical predictability (Chandrasekaran et al., 2009; Giroud et al., 2024; Iverson & Song, 2024). Likewise, nearby voxels or electrodes also tend to share activity due to spatial coupling (Chang et al., 2010; Hullett et al., 2016; Pasley et al., 2012). Neglecting these correlations can lead to incorrect attribution of effects across variables or regions. The second is **temporal dependence**: both current linguistic and neural states are shaped by their own past, reflecting sequential constraints in language and memory- or prediction-based processes in the brain. In language processing, the brain’s response to a word reflects not only its acoustic and lexical features (Heilbron et al., 2022; Hovsepyan et al., 2020) but also preceding context, memory, and expectation (Gow & Caplan, 2012).

We focus on these two dimensions because they correspond to the two forms of structure that are intrinsic to both linguistic input and neural dynamics. Linguistic signals are organized by systematic co-occurrence patterns and sequential dependencies, from phonotactics to semantics, while neural activity expresses analogous organization through momentary population co-activation and history-dependent processes such as recurrence, prediction, and adaptation. Because these parallels shape both what the brain receives and how it responds, any model of language-brain mappings must decide whether to represent, approximate, or ignore these dependencies. Covariance and temporal dependence, therefore, define the two axes that most directly constrain the kinds of inferences different models can support.

These two dependencies can be formalized using a general representation of linguistic predictors and neural responses as time-indexed vectors. Let $X_{t}=\;[x_{1}\left(t\right),\;\ldots ,\;$ $x_{M}\left(t\right){]}^{t}$ and $Y_{t}=\;{\left[y_{1}\left(t\right),\ldots ,y_{N}\left(t\right)\right]}^{T}$ denote two sets of variables at time t, each of which may correspond to linguistic features or neural signals depending on the modeling setup. For notational convenience, we refer to $X_{t}$ as the predictor set and $Y_{t}$ as the predicted set. A general formulation that encompasses a wide range of models is

$$X_{t}=AF(X_{t^{\prime }<t}),\;\;\;CG(Y_{t})=BH(X_{t}).$$

Here, $F(X_{t^{\prime }<t})=\;{\left[f_{1}(X_{t^{\prime }<t}),\;\ldots ,\;f_{M}(X_{t^{\prime }<t})\right]}^{T}$ expresses how past states can be transformed; $G(Y_{t})$ and *$H(X_{t})$* are transformations of the present state; $A\in {\mathbb{R}}^{M\times M}$ controls how past states influence the present; $B\in {\mathbb{R}}^{L\times M}$ and $C\in {\mathbb{R}}^{L\times N}$ define the instantaneous mapping between $X_{t}$ and $Y_{t}$. Here, M and N denote the dimensionality of the predictor and response spaces (i.e., the number of variables in $X_{t}$ and $Y_{t}$). The integer L specifies the dimensionality of the intermediate representational space used to connect the two sets: it may match N in simple observed-state models or be lower or higher in models that introduce a latent space or a representational transformation. This notation is intentionally general: $X_{t}$ may represent linguistic variables and $Y_{t}$ neural responses, or the reverse, depending on the modeling direction.

Temporal dependence is quantified by the model’s reliance on past states: $X_{t}=AF(X_{t^{\prime }<t})$ . The norm $||A|{|}^{2}$ provides a compact measure of the strength of past-to-present influence. When A=0 , the model is static; when A≠0 , the model is dynamic, with larger norms corresponding to stronger temporal dependencies.

Instantaneous dependencies among predictors are summarized by the covariance matrix S=〈 (H− 〈H〉)(H− $\langle H\rangle {)}^{T}\rangle$. The strength of covariance is given by the norm of the off-diagonal entries: ${\left\|S\right\|}^{2}-\sum_{i}{\left|S_{ii}\right|}^{2}$. Small values indicate near-independence among variables; large values indicate substantial shared structure.

**Fig. 3.**
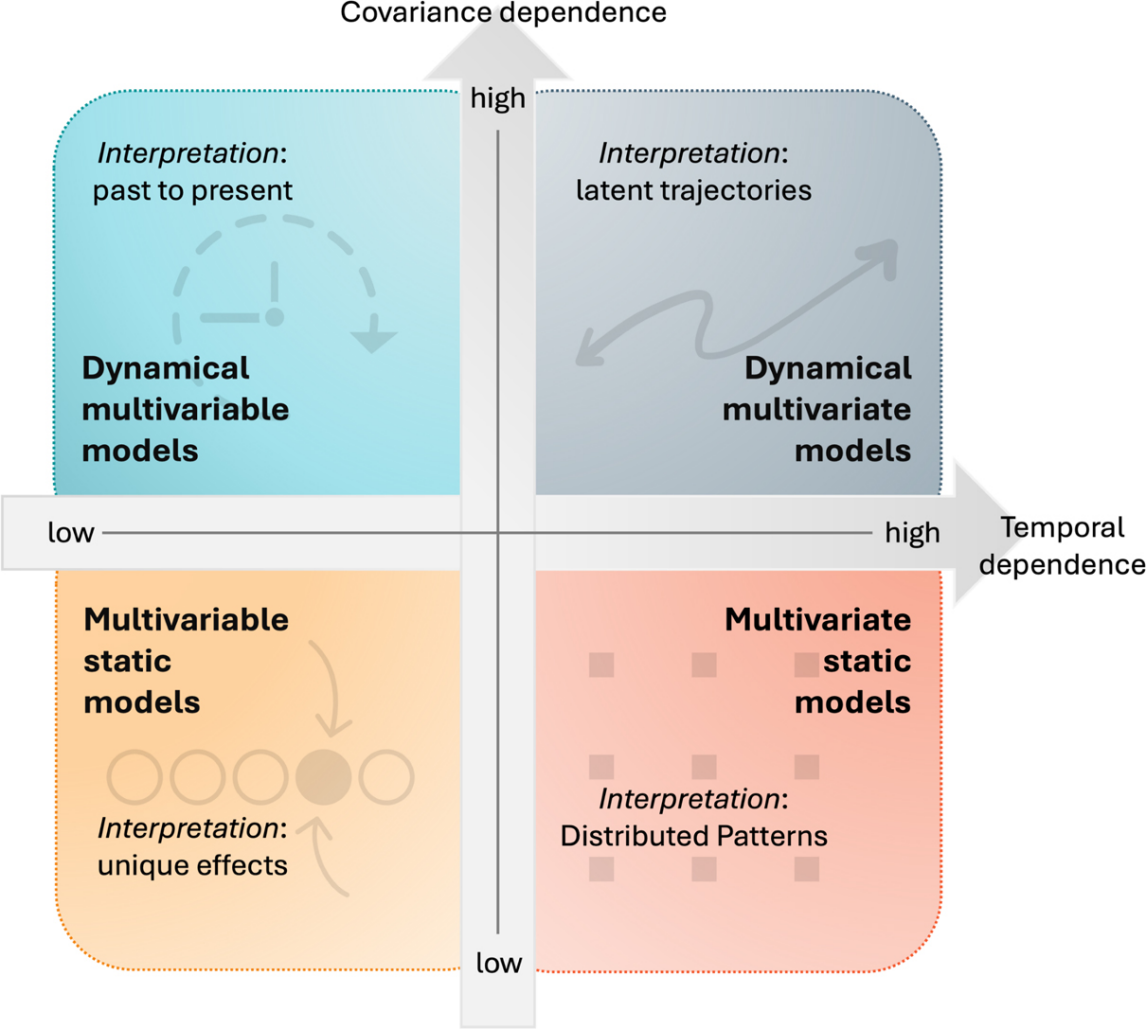
The 2D-taxonomy situates modeling approaches according to two statistical dependencies: instantaneous covariance dependence, illustrated by the relationship $CG(Y_{t})=BH(X_{t})$ , which indicate whether instantaneous shared variance across linguistic or neural outputs is modeled (horizontal axis); and temporal dependence, expressed as $X_{t}=AF(X_{t^{\prime }<t})$ , which indicate whether current linguistic and neural states depends on their own past (vertical axis). The combination of these axes defines four modeling logics, each enabling a distinct type of inference about language-brain relationships:
**Multivariable static models** (e.g., general linear, analyse of variance, and other regression-based approaches) minimize covariance dependencies by assuming that each predictor contributes independently to the modeled signal. They are preferred for hypothesis-driven, deductive inference because their assumptions make effects relatively unambiguous to interpret.**Multivariate static models** (e.g., multiple regression with correlated predictors, partial least squares, representational similarity, or principal component approaches) exploit covariance among predictors to uncover interactions, co-activations, or distributed structures typical of naturalistic language data. They are powerful for pattern discovery but often obscure the unique contribution of individual predictors.**Dynamical multivariable models** (e.g., autoregressive, vector autoregressive, and Granger-causal approaches) explicitly capture how neural activity evolves over time using observable variables such as lagged responses or cross-lags. They enable the study of temporally structured processes and are interpretable when hypotheses concern variations or transitions rather than static contrasts.**Dynamical multivariate models** (e.g., state-space, hidden Markov, or variational latent-dynamical approaches) integrate both covariance and temporal dependencies through latent variables that summarize high-dimensional, correlated, and time-varying data. They uncover hidden dynamics and evolving internal states, but often at the cost of interpretability.

The combination of instantaneous covariance (off-diagonal structure of S) and temporal dependence ($\left||A|{|}^{2}\right)$ defines the two axes of the 2D-taxonomy (Fig. 3). Their intersection yields four model families, each characterized by a distinct inferential logic.

- **Multivariable static models** occupy the low-covariance, low-temporal quadrant. They aim to minimize covariance dependencies by experimental design or by orthogonalizing predictors, and do not model temporal evolution, allowing clear attribution of variance to named factors.
- **Multivariate static models** lie in the high-covariance, low-temporal quadrant. They deliberately represent share variability across features or neural sites, revealing distributed or overlapping structure in language and brain activity.
- **Dynamical multivariable models** correspond to the low-covariance, high-temporal quadrant. They make temporal dependencies explicit, through autoregressive terms, which describe directed temporal influence among signals while maintaining an assumption of instantaneous independence.
- **Dynamical multivariate models** occupy the high-covariance, high-temporal quadrant. They combine temporal evolution with correlated structure, often through latent or state-space formulations that integrate shared variability and memory-based processes.

These four model families provide complementary perspectives on language-brain mapping. Situating modeling approaches within a common dependency space makes the trade-offs associated with each analytical strategy transparent and, we believe, helps improve the readability of the resulting inferences. To make this connection explicit, the next sections turn to the empirical landscape. We review each family, outlining the kinds of findings they have produced in speech and language neuroscience and the aspects of language–brain organisation they have helped uncover.

### 2.1 Multivariable static models: Isolating functional contributions under independence assumptions

Multivariable static models formalize the logic of controlled inference: they enable researchers to attribute variance in one set of variables to specific factors in the other by ensuring that predictors are statistically or experimentally independent. Within the 2D-taxonomy, they occupy the *low-temporal*, *low-covariance* quadrant: neither linguistic or neural history is modeled, and any instantaneous shared variance across linguistic features or neural sites remains unmodeled. Each predictor dimension and each response unit is modeled separately, so that effects can be interpreted as the unique contribution of each factor. This independence logic supports functional attribution, regardless of the mapping direction: when variables are well controlled and minimally correlated, relations between linguistic and neural measures can be interpreted in a straightforward and transparent manner.

This logic underpins the design of tightly structured experimental paradigms, such as factorial or contrast-based designs, where variables are orthogonalized to ensure interpretability. These paradigms operationalize the assumption of independence through explicit experimental control and selective manipulation of linguistic variables. Although this framework is best suited for controlled conditions, it can be extended to naturalistic data if the degree of predictor interdependence is explicitly assessed and interpreted accordingly.

Historically, multivariable static models have provided the statistical backbone of classical, hypothesis-driven approaches in the neuroscience of language. They revealed that linguistic information is hierarchically organized, from acoustic to semantic levels, and that these levels engage partially distinct cortical networks. Studies manipulating low-level acoustic features, such as spectral complexity or modulation rate, have consistently shown activation in early auditory areas, particularly in Heschl’s gyrus (Giroud et al., 2020, 2024; Overath et al., 2012). When higher-order linguistic features are targeted (i.e., lexical frequency, semantic ambiguity, or syntactic embedding), activation shifts to more anterior and inferior regions, including the middle temporal gyrus and inferior frontal cortex (Friederici et al., 2000; Hauk et al., 2006). Together, these findings revealed a graded organization of linguistic abstraction along posterior-anterior and dorsal-ventral pathways (Hagoort & Indefrey, 2014), consistent with hierarchical and dual-stream accounts of language processing (Poldrack et al., 1999; Saur et al., 2008; Visser et al., 2012).

The same inferential logic extends to continuous speech through temporal response functions, which estimate how time-lagged acoustic or linguistic features relate to neural activity using linear regression, without positing internal neural dynamics. This approach maintains the assumptions of the multivariable static quadrant: each site is fit separately, covariance across predictors or neural sites is not modeled, and temporal structure arises only from stimulus-aligned lags. Despite these simplifications, studies using temporal response functions have revealed orderly temporal integration windows across auditory cortex, from millisecond-scale acoustic encoding to longer windows aligning with syllabic, lexical, or even phrasal units (Bai et al., 2022; Barchet et al., 2025; Holdgraf et al., 2016; Venezia et al., 2019).

Across these dimensions of language processing (e.g., hierarchical abstraction, stream dissociation, and graded temporal integration), multivariable models provide a rigorous and interpretable framework for linking linguistic features to neural responses. The inferential clarity of multivariable static models stems from what they exclude: by minimizing covariance and ignoring temporal dependencies, they enable strong attribution of function (Kraus et al., 2024). The cost, however, is potential underfitting in naturalistic settings, where linguistic predictors co-vary and neural activity is structured across both space and time. In such cases, diagnostic analyses often motivate moving rightward (toward multivariate static models) or upward (toward dynamical multivariable or dynamical multivariate models) within the taxonomy.

### 2.2 Multivariate static models: Revealing distributed representations through shared structure

Multivariate static models formalize the logic of shared structure: rather than isolating independent contributions, they capture covariance among predictors and neural responses at a given time. In the 2D-taxonomy, they occupy the high-covariance, low-temporal quadrant: they do not model neural or linguistic history but explicitly represent correlated variability across linguistic features and neural dimensions (Kriegeskorte & Kievit, 2013). This shift in modeling logic moves inference from individual predictors to population-level patterns (Mitchell et al., 2008). By detecting share variance, multivariate static models reveal distributed representations, that are patterns of joint activation that cannot be attributed to any single variable or region.

Technically, these models encompass a wide range of statistical approaches designed to exploit covariance structure: multiple regression with correlated predictors, partial least squares, representational similarity analysis (RSA), principal component analysis (PCA), and related dimensionality-reduction or representational frameworks. Unlike multivariable static models, which treat predictor correlation as confound, multivariate static models treat it as informative structure (i.e., something to be explained rather than removed). They are particularly suited to the complexity of natural language, where linguistic and neural dimensions are inherently interdependent (de Heer et al., 2017; Orepic et al., 2023; Rutten et al., 2019).

When contextualized language models (LMs), including transformer-based or autoregressive variants, are used as feature extractors, their embedding spaces provide rich multivariate predictors. These embeddings, indeed, carry linguistic history, but this history reflects the architecture of the LM, not temporal dependence in the neural model. In the 2D-taxonomy, such approaches remain non-dynamical because the neural response at time t is predicted solely from the current embedding, not from the model’s own past neural states (Dupré La Tour et al., 2022; Jalouzot et al., 2025). In this feature-space role, contextualized representations predict brain activity remarkably well: model-brain correspondence increases with next-word prediction performance and tracks comprehension, especially when embeddings integrate longer context (Caucheteux et al., 2022; Schrimpf et al., 2021).

Multivariate static models have become central for understanding how speech and language are represented in the brain under naturalistic conditions. Studies applying multivariate analyses have shown that acoustic, phonetic, and syntactic information are jointly represented across overlapping cortical networks (Carota et al., 2021; Cope et al., 2023; De Angelis et al., 2018; Zheng et al., 2025), including the superior temporal sulcus, middle temporal gyrus, and inferior frontal cortex (Caucheteux et al., 2022; Chalas et al., 2022; Daube et al., 2019). These results support the view that language is encoded in distributed patterns rather than strictly segregated pathways (Degano et al., 2024; Gwilliams et al., 2022; Huth et al., 2016; Keshishian et al., 2023), supporting a graded, overlapping organization consistent with the dual-stream and hierarchical accounts of language processing. This framework, thus, provides an ecologically grounded view of linguistic computations as a system of interacting representations distributed across multiple spatial and representational scales.

The interpretive cost of this flexibility is loss of specificity. Because variance is shared across predictors and recording sites, multivariate models do not isolate unique effects, and functional attribution becomes weaker (Huth et al., 2016). This limitation is particularly salient in analyses relying on representational spaces, whether derived from PCA, factor analysis, or deep neural embeddings. These approaches have revealed continuous, low-dimensional gradients spanning temporo-parietal and anterior temporal regions (Caucheteux et al., 2022; Zhang et al., 2020). Yet the recovered dimensions can lack clear cognitive or neuroanatomical interpretation (Guest & Love, 2017). Their explanatory value often depends on independent validations (Brendel et al., 2011; Halai et al., 2020; Naselaris & Kay, 2015)—for example, testing whether a derived dimension corresponds to lexical, semantic, or syntactic structure—or controlled manipulations, such as disrupting word order in narratives to assess whether the organization of neural responses reflects meaning only when comprehension is preserved (Broderick et al., 2022).

### 2.3 Dynamical multivariable models: Uncovering history dependence in observed signals

Dynamical multivariable models formalize the logic of temporal dependence: they make current linguistic and neural states explicit functions of their respective pasts, allowing the study of how both systems evolve and influence one another over time. In the 2D-taxonomy, they occupy the high-temporal, low-covariance quadrant. These models incorporate autoregressive, cross-lag, or history terms that link present responses to lagged activity, while instantaneous shared variance across features or recording sites remains unmodeled. The temporal coordinate of a model increases only when the present depends explicitly on its own history, whether linguistic, neural, or both. This logic shifts inference from static structure to process and rather than identifying where linguistic features are represented, it characterizes how encoding unfolds dynamically across time.

Dynamical multivariable approaches include autoregressive (AR) and vector-autoregressive (VAR) models, which remain underused in speech and language neuroscience despite being the natural tools for explicitly temporal questions (i.e., whether past states in either linguistic or neural domain contribute to predicting current neural activity). In multichannel recordings during speech perception, VAR models link each time series to its own and other regions’ lagged activity, revealing coordinated interactions between sensory and association cortices (Goebel et al., 2003). Within this framework, Granger causality provides a statistical basis for inferring directed influence among observable sources by testing whether the past of one signal improves prediction of another.

Applied to speech and language, dynamical multivariable analyses have shown that slow fluctuations in association auditory regions modulate gamma-band responses in primary auditory areas during sentence comprehension, reflecting contextual shaping of early acoustic encoding (Fontolan et al., 2014). Similarly, beta-band activity in frontal cortices has been found to modulate gamma responses in the superior temporal gyrus during phoneme categorization (Bouton et al., 2018; Gow et al., 2008), consistent with top-down predictive feedback (van Wassenhove, 2013). These findings illustrate how recurrence transforms abstract notions of “feedforward” and “feedback” into testable temporal dependencies between cortical regions.

The temporal logic of these models also helps clarify the relationship between the dynamics of speech and those of neural activity. Speech is a continuous acoustic signal that must be segmented into discrete linguistic units (e.g., phonemes, morphemes, words) that unfold sequentially and constrain one another over time. Models that capture such linguistic regularities treat the input itself as temporally structured: Markov chains and recurrent neural networks (RNNs), for instance, encode dependencies between past and current linguistic states, from short-range transitions to longer syntactic or semantic relations. These architectures enrich the predictor space by embedding contextual information at each time point of the linguistic input (Gales & Young, 2007; Lakretz et al., 2021; Moses et al., 2016).

A central challenge for dynamical modeling lies in scaling these methods to naturalistic, high-dimensional data. Classic VAR and Granger methods tend to yield unstable or opaque interaction graphs when applied to large datasets. Regularized extensions such as Sparse VAR (Krampe & Paparoditis, 2021; Mody & Rangarajan, 2022) and conditional Granger analysis (Y. Chen et al., 2006; Z. Zhou et al., 2009) mitigate this issue by pruning redundant connections and controlling for common inputs, yielding more reliable and interpretable network-level inferences. Still, because all variables need to be observable, these models cannot capture latent processes that jointly drive observed activity. Addressing this limitation requires moving upward in the taxonomy—to *dynamical multivariate models*, which introduce hidden states to infer the internal trajectories that organize time-varying neural and linguistic representations.

### 2.4 Dynamical multivariate models: Inferring evolving representations through latent dynamics

Dynamical Multivariate models ask how a latent state evolves over time and mediates the mapping between linguistic input and neural activity. In the 2D-taxonomy, they occupy the high temporal, high-covariance quadrant because the latent state carries information from one moment to the next and reflects covariance in the linguistic input or the neural responses. The model integrates these two sources of dependence rather than treating them as noise or confounds. Even when noise is assumed to be independent across recording sites, shared dependence on the latent state induces instantaneous covariance among neural signals, because multiple sites express different projections of a common underlying process. Likewise, linguistic predictors that co-occur in natural speech can be mapped jointly into the latent state, so that correlations in the stimulus space explicitly shape the trajectory the latent state follows over time. By allowing both linguistic and neural covariance structures to influence the same evolving hidden process, dynamical multivariate models treat temporal and instantaneous dependencies as jointly generated by the latent dynamics. In this framework, the temporal evolution of the latent state produces not only the time-varying structure of the neural response but also the way correlated linguistic cues jointly shape that evolution. The temporal and covariational structure observed in the data, therefore, emerges from a single, unified generative mechanism linking linguistic input and neural activity over time.

The core assumption is that high-dimensional observed signals are projections of a lower-dimensional, time-evolving latent process that integrates prior internal states with external input (Miller, 2016). Canonical approaches such as Hidden Markov Models (HMMs), state-space models (SSMs), and Gaussian Process Latent Variable Models (GPLVMs) implement this principle by estimating hidden trajectories and their uncertainty from complex time series. Although these models differ in how observation noise is specified, they all enforce a multivariate structure through the shared latent state, which produces coordinated patterns across variables even in the absence of an explicit full covariance matrix. Their validity depends on identifiability constraints and predictive checks verifying that the inferred dynamics account for observed auto- and cross-covariances. More flexible extensions, including variational and nonlinear state-space models, allow for richer relationships between hidden and observed variables. Autoregressive transformer language models embody a similar logic by maintaining evolving contextual representations, albeit without explicitly parameterizing latent states (Goldstein et al., 2022).

Applied to brain-language mapping, dynamical multivariate models have revealed hidden temporal organization linking linguistic representations and neural activity. They recover internal state sequences aligned with phonemic or lexical units (Li et al., 2023), track attentional shifts in multi-speaker scenes (Akram et al., 2016), and identify continuous trajectories consistent with hierarchical linguistic structure (Moses et al., 2016). Converging evidence suggests that speech-evoked activity operates across nested temporal scales, with faster latent dynamics encoding phonemic information and slower trajectories reflecting lexical or conceptual integration (Donhauser & Baillet, 2020). In anterior superior temporal regions, latent trajectories capture converging bottom-up and top-down influences consistent with real-time integration of acoustic and semantic cues (Hamilton et al., 2018). Wave-like propagations across auditory cortex preceding word onsets indicate anticipatory tuning and predictive pre-activation (Friston, 2019; Muller et al., 2018; Pang (庞兆阳) et al., 2020; Stephen et al., 2023). In this view, discrete linguistic units such as phonemes and words emerge as transient condensations within a continuously evolving low-dimensional space, suggesting that discrete percept arises from smooth neural dynamics (Medrano et al., 2024; Orepic et al., 2023).

The interpretive cost, however, is substantial. Because latent spaces are not directly observed, their dimensionality and geometry inherit model assumptions that can bias conclusions (Humphries, 2021). Too few latent dimensions risk collapsing distinct processes, while too many entails overfitting and poor generalization. These risks are particularly relevant when mapping trajectories onto specific computations, such as syntactic parsing, prediction, or attentional control (Tavoni et al., 2022). Emerging techniques such as variational inference, nonlinear state-space models, and manifold-aware priors recover richer structures (Aghagolzadeh & Truccolo, 2014; D. Zhou & Wei, 2020; Klindt et al., 2021; Sani et al., 2021; Schneider et al., 2023), but to support explanatory claims they must be accompanied by biologically grounded constraints, explicit identifiability assumptions, transparent uncertainty estimation, and posterior-predictive checks. With these safeguards, latent dynamical models can provide explanatory accounts of internal, time-evolving structure; without them, they are best treated as tools for hypothesis generation and representational mapping.

These empirical examples make clear that each family illuminates a different facet of the language–brain relationship (Table 1). To bring these contrasts into focus, the following section presents a single running example: a common experimental scenario examined through the lens of all four model families, revealing how each one, through the dependencies it represents, supports a different interpretation.

**Table 1.** Overview of model classes in the Dual-Dependency Taxonomy, summarizing their assumptions, strengths, limitations, and typical applications in speech and language neuroscience.

Model category	Key characteristics	Strengths	Limitations	Examples of use in speech & language neuroscience
Multivariable static models	Model effects of individual predictors assumed to act independently	High interpretability and clear attribution of neural responses to specific linguistic features; ideal for hypothesis testing	Assumes minimal covariance; limited ecological validity; may underfit complex or naturalistic data	Encoding of acoustic–linguistic hierarchies (Heschl’s gyrus → IFG); dual-stream dissociations; temporal response function in controlled settings
Multivariate static models	Model shared variance and distributed structure across predictors or brain regions	Sensitive to co-activation and interaction effects; suited for high-dimensional data; reveals population-level structure	Limited functional specificity; emergent dimensions may lack cognitive interpretability; inference remains correlational	Distributed phonetic–semantic representations in STS and MTG; semantic gradients across cortex; alignment between model and brain representational spaces
Dynamical multivariable models	Model explicit temporal dependencies among observed signals over time, while assuming instantaneous independence	Capture sequential processing and feedback; make directed temporal influences testable; compatible with predictive coding frameworks	Limited spatial scale; do not infer hidden states; may overfit in high-dimensional data	Temporal coordination in speech perception; top-down modulation of auditory activity; cross-frequency feedback during phoneme categorization
Dynamical multivariate models	Model hidden internal states that evolve over time and jointly explain temporal dependencies and instantaneous covariance	Capture nested timescales and abstract internal structure; reveal low-dimensional latent trajectories underlying neural dynamics	Lower interpretability; results depend on model assumptions and identifiability; risk of overfitting	Phoneme-to-word transitions as latent trajectories; anticipatory dynamics in auditory cortex; hierarchical linguistic integration

### 2.5 A running thought experiment: How different models answer different scientific questions

To illustrate how the four model families differ in the dependencies they represent and the inferences they support, we consider a deliberately minimal scenario. A listener hears one of two short sentence contexts. One context strongly biases the perception of **knight** (“Every morning, the villagers gathered as the knight approached the gate”), whereas the other favors the perception of **night** (“Every evening, the villagers gathered as the night settled over the valley”). To create a controlled phonological uncertainty, we replace the natural homophony of these words with a morphed onset halfway between /kn/ and /n/. This ambiguous token is identical across conditions, so any neural difference at the moment of ambiguity must arise from contextual (lexical or semantic) rather than acoustic influences.

We represent the linguistic variables Y(t) using a single component: lexical expectation. This variable quantifies the probability of the upcoming word given the sentence context, estimated either from human cloze norms or predictive language models. It differs sharply between the knight-biased and night-biased contexts. For the purpose of this thought experiment, lexical expectation is treated as a static variable at $t_{0}$: although its antecedent cues accumulate over the sentence, their temporal evolution is not explicitly modeled on the linguistic side in this example. Any anticipatory influence it exerts before $t_{0}\;$ must, therefore, appear indirectly through the dynamics of the neural signal. Neural activity is recorded from primary auditory cortex (A1), posterior superior temporal gyrus (pSTG), and anterior temporal lobe (ATL), forming a multichannel time series X(t).

We could use **multivariable static models** to ask whether the lexical expectation at the moment of ambiguity can be decoded from the instantaneous neural response, given that the acoustic input at $t_{0}$ is identical across conditions. Formally, $Y_{lexical}(t_{0})=\beta \text{X}(t_{0})+\varepsilon$ . Because only the lexical variable varies across trials, inference is purely attributional: does the neural snapshot at *t*_0_ carry enough information to distinguish the contextually biased percept? By ignoring temporal dependencies and fitting each neural channel separately, these models isolate which neural sites carry information about the expected percept, revealing the locus of context-driven bias.

**Multivariate static models** could instead ask whether a distributed neural pattern at $t_{0}$ encodes lexical expectation. Here, lexical expectation is decoded from the multiregional activity vector, $Y(t_{0})=BX(t_{0})+\varepsilon$ . Successful decoding shows that contextual bias is manifested in a multiregional population pattern spanning A1, pSTG and ATL, rather than by separable regional contributions. These models explicitly exploit covariance across neural channels and, therefore, probe representational structure, not unique feature effects in individual regions.

Using **dynamical multivariable models**, we can incorporate temporal dependence and ask, for example, whether neural history enhances decoding of lexical expectation at $t_{0}$: $Y_{\text{lexical}}(t_{0})=\sum_{\ell \in ℒ}\beta_{\ell \text{​}}X(t_{0}-\ell )+\varepsilon$ . Here, inference concerns how neural evidence accumulates over time: does activity in earlier time windows help predict which word the listener expects? Because each neural feature and lag enters as an independent predictor, covariance between neural channels is still not represented explicitly. These models reveal whether pre-activation or anticipatory processes shape neural dynamics before the ambiguous token is heard.

Finally, **dynamical multivariate models** could examine whether the multiregional neural trajectory prior to $t_{0}$ predicts lexical expectation when treated as a distributed pattern evolving over time: $Y(t_{0})=\sum_{\ell \in ℒ}B_{\ell }X(t_{0}-\ell )+\varepsilon .$ This formulation encodes both temporal dependence (through the lags) and instantaneous covariance (through the multichannel patterns at each lag). In this minimal scenario, by expressing temporal structure directly through lagged multiregional predictors, this approach tests whether the unfolding population response approaching the ambiguous token is sufficiently organized in space and time to support decoding of the expected word.

## 3 Implications for Model Selection, Evaluation, and Scientific Inference

The Dual-Dependency Taxonomy organizes model choice according to two fundamental structures in language-brain mapping, allowing to clarify how evidence must be evaluated. Each model family embodies specific assumptions about covariance and temporal dependence, and these assumptions determine the kinds of validation required for scientific inference. A model can only answer the questions it is structurally equipped to represent, and its conclusions are trustworthy only if the relevant dependencies are evaluated appropriately.

For models aimed at functional attribution, such as multivariable static approaches, interpretability relies on the precision of estimated effects. Reporting confidence intervals or other uncertainty measures is, therefore, essential for assessing the credibility of factor-specific contributions (G. Chen et al., 2017). For models that seek to reveal distributed representational structure, including multivariate static methods, evaluation focuses on the stability and reliability of the recovered patterns. Robustness across datasets and comparisons to noise ceilings are necessary to determine whether shared variance genuinely reflects linguistic-neural structure (Helmer et al., 2024). For dynamical multivariable models, one must respect temporal order. Cross-validation procedures should be designed so that inferred directed influences cannot arise from statistical leakage across time, but reflect genuine temporal dependencies in the data (Cerqueira et al., 2020). Dynamical multivariate models, which introduce latent states, require additional safeguards. Posterior predictive checks are needed to verify that inferred latent trajectories reproduce the temporal and covariational organization of the data, rather than reflecting modeling assumptions or overly strong priors (Schneider et al., 2023). Together, these evaluation regimes anchor each model family to the dependencies it is meant to represent. They turn the taxonomy into a practical framework for aligning inferential claims with methodological rigour, ensuring that interpretability is demonstrated rather than assumed.

## 4 Concluding Remarks

As the field increasingly relies on large scale datasets and complex, deep modeling architectures, the tension between predictive performance and interpretability becomes central. Researchers across disciplines face a widening divide between algorithmic discovery and human understanding. Two decades ago, the writer Ted Chiang imagined a world in which enhanced “metahuman” scientists produced knowledge too complex for ordinary humans to comprehend (Chiang, 2000). His metaphor feels prescient in the age of artificial intelligence: today’s models generate remarkably accurate predictions, yet their inner logic often remains opaque. Chiang’s vision also carries a hopeful inversion in which the scientist’s role is not to outpace the machine but to ask the right questions, to define relevance, and to frame the purpose of knowledge itself. Beyond serving as a decisional guide, the 2D taxonomy is meant as a tool for reflection. It helps sustain a dialogue between inductive modeling and deductive reasoning, promoting clarity of inference.

## Data Availability

No new data or code were generated for this review article.
